# Frequent somatic loss of *BRCA1* in breast tumours from *BRCA2* germ-line mutation carriers and vice versa

**DOI:** 10.1054/bjoc.2001.2062

**Published:** 2001-10

**Authors:** S Staff, J J Isola, O Johannsson, Å Borg, M M Tanner

**Affiliations:** Laboratory of Cancer Genetics, Institute of Medical Technology, University Hospital of Tampere, University of Tampere, FIN-33014, Finland; Department of Oncology, University Hospital, Lund, S-221 85, Sweden

**Keywords:** *BRCA1*, *BRCA2*, allelic imbalance, LOH, FISH

## Abstract

Breast cancer susceptibility genes *BRCA1* and *BRCA2* are tumour suppressor genes the alleles of which have to be inactivated before tumour development occurs. Hereditary breast cancers linked to germ-line mutations of *BRCA1* and *BRCA2* genes almost invariably show allelic imbalance (AI) at the respective loci. BRCA1 and BRCA2 are believed to take part in a common pathway in maintenance of genomic integrity in cells. We carried out AI and fluorescence in situ hybridization (FISH) analyses of *BRCA2* in breast tumours from germ-line *BRCA1* mutation carriers and vice versa. For comparison, 14 sporadic breast tumours were also studied. 8 of the 11 (73%) informative *BRCA1* mutation tumours showed AI at the *BRCA2* locus. 53% of these tumours showed a copy number loss of the *BRCA2* gene by FISH. 5 of the 6 (83%) informative *BRCA2* mutation tumours showed AI at the *BRCA1* locus. Half of the tumours (4/8) showed a physical deletion of the *BRCA1* gene by FISH. Combined allelic loss of both *BRCA1* and *BRCA2* gene was seen in 12 of the 17 (71%) informative hereditary tumours, whereas copy number losses of both *BRCA* genes was seen in only 4/14 (29%) sporadic control tumours studied by FISH. In conclusion, the high prevalence of AI at *BRCA1* in *BRCA2* mutation tumours and vice versa suggests that somatic events occurring at the other breast cancer susceptibility gene locus may be selected in the cancer development. The mechanism resulting in AI at these loci seems more complex than a physical deletion.   http://www.bjcancer.com © 2001 Cancer Research Campaign


*British Journal of Cancer* (2001) **85**(8), 1201–1205
© 2001 Cancer Research Campaign
doi: 10.1054/ bjoc.2001.2062, available online at http://www.idealibrary.com on IDEAL

### Frequent somatic loss of *BRCA1* in breast tumours from *BRCA2* germ-line mutation carriers and vice versa

**S Staff[1], JJ Isola[1], O Johannsson[2], Å Borg[2] and MM Tanner[1]**

[1]Laboratory of Cancer Genetics, Institute of Medical Technology, University Hospital of Tampere, FIN-33014 University of Tampere, Finland;
[2]Department of Oncology, University Hospital, S-221 85 Lund, Sweden

**Summary** Breast cancer susceptibility genes *BRCA1* and *BRCA2* are tumour suppressor genes the alleles of which have to be inactivated before tumour development occurs. Hereditary breast cancers linked to germ-line mutations of *BRCA1* and *BRCA2* genes almost invariably show allelic imbalance (AI) at the respective loci. BRCA1 and BRCA2 are believed to take part in a common pathway in maintenance of genomic integrity in cells. We carried out AI and fluorescence in situ hybridization (FISH) analyses of *BRCA2* in breast tumours from germ-line *BRCA1* mutation carriers and vice versa. For comparison, 14 sporadic breast tumours were also studied. 8 of the 11 (73%) informative *BRCA1* mutation tumours showed AI at the *BRCA2* locus. 53% of these tumours showed a copy number loss of the *BRCA2* gene by FISH. 5 of the 6 (83%) informative *BRCA2* mutation tumours showed AI at the *BRCA1* locus. Half of the tumours (4/8) showed a physical deletion of the *BRCA1* gene by FISH. Combined allelic loss of both *BRCA1* and *BRCA2* gene was seen in 12 of the 17 (71%) informative hereditary tumours, whereas copy number losses of both *BRCA* genes was seen in only 4/14 (29%) sporadic control tumours studied by FISH. In conclusion, the high prevalence of AI at *BRCA1* in *BRCA2* mutation tumours and vice versa suggests that somatic events occurring at the other breast cancer susceptibility gene locus may be selected in the cancer development. The mechanism resulting in AI at these loci seems more complex than a physical deletion. © 2001 Cancer Research Campaign http://www.bjcancer.com

**Keywords**: *BRCA1; BRCA2*; allelic imbalance; LOH; FISH

Approximately 5–10% of breast cancer is due to inherited predisposition (Miki et al, 1994). Germ-line mutations in the two identified susceptibility genes, *BRCA1* (Miki et al, 1994) and *BRCA2* (Wooster et al, 1994; Tavtigian et al, 1996) are responsible for a large proportion of hereditary breast cancer (Szabo and King, 1997). Both *BRCA1* and *BRCA2* are considered as classical tumour suppressor genes and therefore inactivation of both alleles is required for cancer initiation. Although no sequence homology has been found between *BRCA1* and *BRCA2*, they share many functional properties (reviewed in Welcsh et al, 2000).

Almost all the tumours from germ-line *BRCA1* and *BRCA2* mutation carriers show loss of heterozygosity (LOH) or AI at the corresponding loci (Smith et al, 1992; Neuhausen and Marshall, 1994; Collins et al, 1995; Gudmunsson et al, 1995; Staff et al, 2000), which is in accordance with the lost tumour suppressor function. Due to several functional parallels between *BRCA1* and *BRCA2*, we studied the possible somatic aberrations of *BRCA1* by AI and FISH in breast cancer tumours from germ-line *BRCA2* mutation carriers, and vice versa. The possible concomitant somatic aberrations of the *BRCA1* and *BRCA2* genes were also studied in 14 sporadic breast cancer samples by FISH. We have previously shown (Staff et al, 2000) that unlike in hereditary BRCA1/2 tumours, the allelic imbalance at BRCA1/2 loci is almost always a result of a physical deletion in sporadic tumours. Therefore, physical deletion of the *BRCA* genes detected by FISH reflects the allelic imbalance of the *BRCA1/2* loci in sporadic tumours (Staff et al, 2000).

*Received 2 March 2001*
*Revised 2 July 2001*
*Accepted 2 July 2001*

*Correspondence to:* S Staff, Laboratory of Cancer Genetics, Institute of Medical Technology, Lenkkeilijankatu 6, 33014 University of Tampere, Finland; E-mail: ss59025@uta.fi

### MATERIALS AND METHODS

### Patients and tumour samples

17 primary breast cancer tumours from germ-line *BRCA1* mutation carriers and 8 primary breast tumours from germ-line *BRCA2* mutation carriers were derived from the Department of Oncology, University of Lund. 14 primary sporadic breast cancer tumours were obtained from Tampere University Hospital. The tumour samples were snap-frozen and stored at –70˚C until used for AI and FISH analyses.

Genomic DNA was extracted from available blood samples of the 13 *BRCA1* and 6 *BRCA2* germ-line mutation carriers by standard methods. One *BRCA1* patient had 2 separate tumours (Ca 8571 and Ca 13996; Table 1), which were both analysed. *BRCA1* patients with tumours Ca 14090 and Ca 14007 (Table 1) were relatives, but none of the other *BRCA1* patients were directly related. One *BRCA2* patient had also 2 separate tumours (Ca 11 900 and 14 486; Table 2). *BRCA2* patients with tumours Ca 7936 and Ca 11506 were from the same family, similarly as patients with tumours Ca 11787 and Ca 13816 (Table 2). *BRCA1* and *BRCA2* mutation analyses have been described previously (Johansson et al, 1996; Håkansson et al, 1997; Tables 1 and 2).

### PCR microsatellite analysis

Polymerase chain reaction (PCR) was used to detect AI at polymorphic microsatellite markers by comparing the allelic patterns

**Table 1** Copy number aberrations of *BRCA2* by FISH and AI in 17 breast cancers from germ-line *BRCA1* mutation carriers

| Tumour | BRCA1 mutation | Result of the BRCA1 mutation | AI at the BRCA1 locus[a] | AI at the BRCA2 locus[b] | DNA Index[c] | Mean copy number/cell of BRCA2 (±SEM) | Mean copy number/cell of 13q reference probe (±SEM) | Mean copy number ratio (BRCA2/ 13q reference probe) | Interpretation of the BRCA2 copy number by FISH[d] |
|---|---|---|---|---|---|---|---|---|---|
| Ca 12 421 | 2594delC | Ile845Stop | NA | NA | 1.56 | 3.84 (0.11) | 2.55 (0.09) | 1.51 | 3:4 *BRCA2* gain |
| Ca 11 808 | 3829delT | Leu1263Stop | Yes | Yes | 1.0 | 1.18 (0.09) | 1.20 (0.09) | 0.98 | Monosomy of 13q |
| Ca 09 252 | 2594delC | Ile845Stop | Yes | Yes | 1.8 | 2.47 (0.11) | 3.45 (0.10) | 0.72 | 3:2 *BRCA2* deletion |
| Ca 14 007 | 3172ins5 | Thr1025Stop | Yes | Yes | 1.53 | 1.43 (0.08) | 2.71 (0.10) | 0.53 | 3:2 *BRCA2* deletion |
| Ca 10 581 | 1806C→T | Gln563Stop | Yes | NI | 1.73 | 2.17 (0.12) | 3.66 (0.16) | 0.59 | 4:2 *BRCA2* deletion |
| Ca 12 224 | 1806C→T | Gln563Stop | Yes | Yes | 1.52 | 2.10 (0.10) | 2.50 (0.11) | 0.84 | Large deletion at 13q[e,f] |
| Ca 14 510 | 300T→G | Cys61Gly | Yes | Yes | 2.46 | 2.43 (0.17) | 2.53 (0.15) | 0.96 | Large 13q deletion[f] |
| Ca 10 360 | 3172ins5 | Thr1025Stop | Yes | Yes | 1.7 | 1.80 (0.09) | 1.94 (0.07) | 0.98 | Large 13q deletion[f] |
| Ca 13 812 | 4808C→G | Glu1115Stop | Yes | NI | 1.87 | 1.82 (0.08) | 1.98 (0.08) | 0.92 | Large 13q deletion[f] |
| Ca 13 714 | 5382insC | Glu1829Stop | Yes | No | 2.48 | 3.30 (0.18) | 3.06 (0.09) | 1.08 | Large 13q deletion[f] |
| Ca 13 996 | 1806C→T | Gln563Stop | Yes | Yes | 1.11 | 1.84 (0.09) | 2.24 (0.10) | 0.82 | No relative copy number change |
| Ca 14 970 | 2594delC | Ile845Stop | Yes | Yes | 1.00 | 2.26 (0.11) | 2.21 (0.09) | 1.02 | No relative copy number change |
| Ca 11 394 | 1177G→A | Trp353Stop | NA | NA | 1.0 | 2.40 (0.14) | 2.95 (0.14) | 0.81 | No relative copy number change |
| Ca 08 822 | 1201del11 | Ser361Stop | Yes | NA | 1.69 | 3.16 (0.16) | 3.35 (0.11) | 0.94 | No relative copy number change |
| Ca 10 697 | Linkage + | | Yes | NA | 1.51 | 3.11 (0.17) | 3.18 (0.15) | 0.98 | No relative copy number change |
| Ca 14 090 | 3172ins5 | Thr1025Stop | Yes | No | 1.00 | 2.08 (0.10) | 2.19 (0.08) | 0.95 | No relative copy number change |
| Ca 08 571 | 1806C→T | Gln563Stop | Yes | No | NA | 3.20 (0.19) | 3.35 (0.17) | 0.96 | No relative copy number change |

Copy numbers represent the mean of at least 50 nuclei counted from each sample. (NA = Not available, NI = Not informative) [a]Previously published (Staff et al, 2000). [b]Allelic imbalance was analysed using microsatellite markers 13S267 and 13S260. AI was stated if at least one of the markers used indicated imbalance (compared to normal DNA) of more than 25% between the alleles in tumour sample. [c]DNA index by DNA flow cytometry. [d]Deletion was defined if the copy number ratio was 0,80 or less. Gain was defined if the copy number ratio was 1.30 or more. [e]3:2 *BRCA2* deletion in a subpopulation. [f]When DNA-index was used as copy number reference, the copy number ratios indicated a large deletion in 13q spanning both *BRCA2* and *ETB* genes. When 13q probe (*ETB*) was used as a reference probe, no *BRCA2* gene copy number change was revealed.

**Table 2** Copy number aberrations of *BRCA1* by FISH and AI in 8 breast cancers from germ-line *BRCA2* mutation carriers

| Tumour | BRCA2 mutation | Result of the BRCA2 mutation | AI at the BR TCA2 locus[a] | AI at the BRCA1 locus[b] | DNA index[c] | Mean copy number/cell of BRCA1 (±SEM) | Mean copy number/cell of chr 17 centromere (±SEM) | Mean copy number ratio (BRCA1/ chr 17 cen) | Interpretation of the BRCA1 copy number by FISH[d] |
|---|---|---|---|---|---|---|---|---|---|
| Ca 11 900 | 2024del5 | Ser599Stop | Yes | Yes | 1.89 | 2.27 (0.15) | 5.44 (0.27) | 0.42 | 5:2 *BRCA1* deletion |
| Ca 10 588 | 4486delG | Val1447Stop | NA | NA | 1.07 | 1.10 (0.04) | 2.0 (0.07) | 0.55 | 2:1 *BRCA1* deletion |
| Ca 13 816 | 3058A→T | Lys944Stop | Yes | Yes | 1.00 | 1.18 (0.05) | 1.0 (0.00) | 1.18 | Monosomy of chromosome 17 |
| Ca 14 486 | 2024del5 | Ser599Stop | Yes | Yes | 1.87 | 2.14 (0.11) | 4.42 (0.20) | 0.48 | 4:2 *BRCA1* deletion |
| Ca 07 936 | 6293C→G | Ser2022Stop | No | Yes | NA | 2.19 (0.13) | 2.34 (0.14) | 0.94 | No relative copy number change |
| Ca 11 721 | 5445del5 | Tyr1739Stop | NA | NA | 1.00 | 3.04 (0.18) | 3.68 (0.13) | 0.83 | No relative copy number change |
| Ca 11 787 | 3058A→T | Lys944Stop | Yes | Yes | 1.94 | 4.08 (0.12) | 3.92 (0.09) | 1.04 | No relative copy number change |
| Ca 11 506 | 6293C→G | Ser2022Stop | Yes | No | 1.96 | 2.77 (0.12) | 2.27 (0.07) | 1.22 | No relative copy number change |

Copy numbers represent the mean of at least 50 nuclei counted from each sample. (NA = Not available). [a]Previously published (Staff et al, 2000). [b]Allelic imbalance was analysed using microsatellite markers 17S1322 and 17S855. AI was stated if at least one of the markers used indicated imbalance (compared to normal DNA) of more than 25% between the alleles in tumour sample. [c]DNA index by DNA flow cytometry. [d]Deletion was defined if the copy number ratio was 0.80 or less. Gain was defined if the copy number ratio was 1.30 or more.

© 2001 Cancer Research Campaign of tumour and blood DNA. Two *BRCA1* intragenic markers (D17S855 and D17S1322) (Albertsen et al, 1994) and 2 markers physically linked to *BRCA2* (D13S260 and D13S267) (Wooster et al, 1994) were analysed using primers with published sequence (Gyapay et al, 1994) (Research Genetics, Huntsville, AL, USA). The PCR reactions were carried out as previously described (Staff et al, 2000). 1 μl of the PCR product was analysed by capillary electrophoresis using ABI PRISM™310 Genetic Analyser and GeneScan 2.1 Software according to the manufacturer's instructions (Perkin-Elmer). For the informative heterozygous markers, the AI was determined by calculating ratio of the alleles (L) as previously described (Staff et al, 2000). If L < 0.75 or L > 1.33, then one of the alleles has decreased more than 25% resulting in AI, as previously defined (Kerangueven et al, 1997).

### FISH analyses

FISH analyses were performed using gene-specific PAC probes for *BRCA1* (PAC 103014) and *BRCA2* (PAC 92M18) genes. The specificity of these clones has previously been confirmed (Staff et al, 2000). Chromosome 17 centromere probe (p17H8) was used as a copy number reference for *BRCA1*. For *BRCA2*, a PAC probe specific for the *ETB* gene (at 13q22) was used as a reference, because specific centromere probe for chromosome 13 is not available. The hybridization efficiency of the probes was tested in a non-malignant breast sample. Hybridization and detection were performed as previously described (Tanner et al, 1998; Staff et al, 2000). Hybridization signals from 50–100 nuclei were scored to assess the copy number of the *BRCA1* and *BRCA2* genes. Deletion of the *BRCA* genes was defined as an average ratio ≤ 0.80 of *BRCA1/2* signals relative to chromosome 17 centromere signals or *ETB* signals, respectively. Gain was defined as an average ratio of ≥1.30. Digital images were taken with a Hamamatsu 9585 camera (Hamamatsu, Hamamatsu City, Japan) operated via ISIS image analysis software (MetaSystems, Altslussheim, Germany).

### RESULTS

### *BRCA1* and *BRCA2* tumours

11 out of 13 *BRCA1* mutation carriers with available blood samples were informative, i.e. they were heterozygous for at least one of the two *BRCA2* markers. AI at *BRCA2* was found in 8 of the 11 (73%) informative cases (Figure 1, Table 1). All the 17 *BRCA1* tumours were analysed for the *BRCA2* gene copy number by FISH. 3 tumours showed a clear physical interstitial deletion of the *BRCA2* gene when *BRCA2* signals were compared to the reference gene signal counts (*ETB* gene at 13q22) (Figure 1, Table 1). If the overall ploidy level (= DNA index by flow cytometry) was used as a *BRCA2* copy number reference, 6 additional tumours showed a loss of *BRCA2*. This suggests a large deletion at 13q comprising both *ETB* and *BRCA2* genes in all but one of these tumours

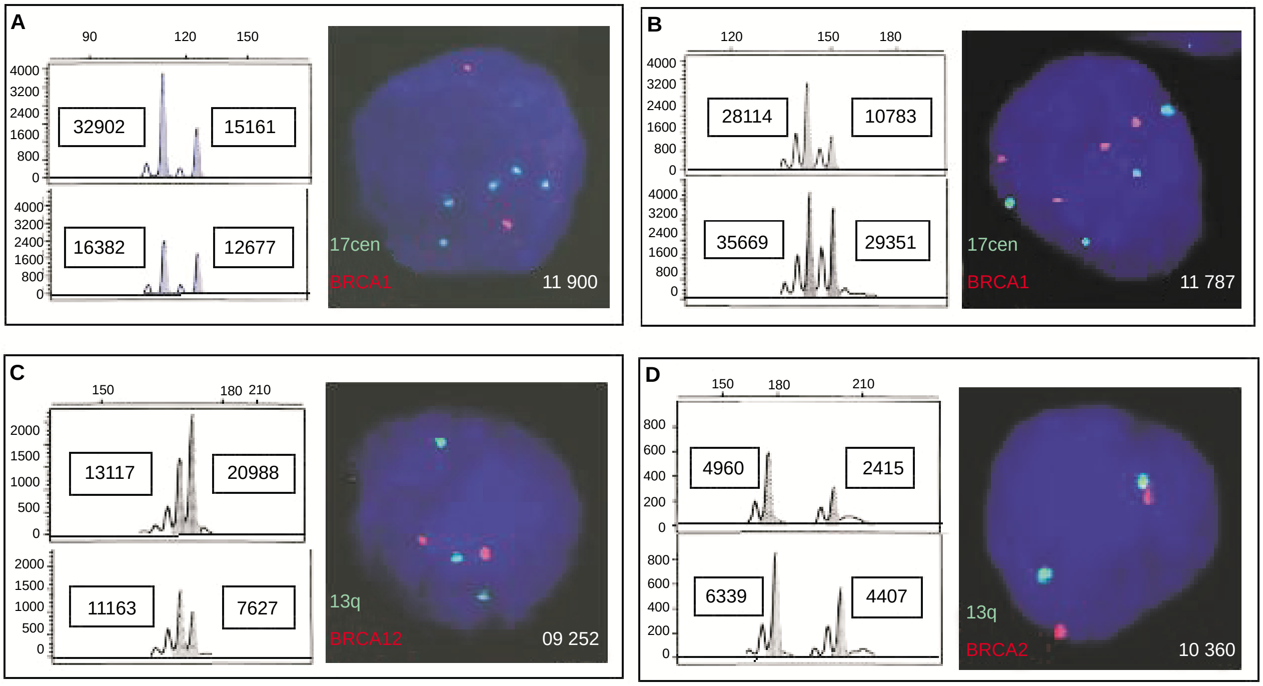

**Figure 1** Examples of the assessment of allelic imbalance (AI) by automated DNA sequencer and two-colour FISH of *BRCA1*, chromosome 17 centromere, *BRCA2* and *ETB* (13q reference probe). The AI and FISH analyses of the same tumour are presented next to each other so that AI analysis is shown in the left. The fragment analysis of PCR products is shown from tumour DNA (top rows) and from matched blood DNA (bottom rows). Size of PCR products (in base pairs) is shown on the X-axis, and the peak heights in fluorescence units are shown on the Y-axis. The alleles in the normal DNA and the corresponding peaks in the tumour DNA are shown in grey. The corresponding allele peak areas in informative tumours are presented in boxes next to the peaks. In FISH images, the probes are visualised in green and red colours (fluorescein and Texas Red, respectively). The probes are marked with the corresponding colour in each panel. The nuclei were counterstained with DAPI (blue). The case numbers are marked in each panel with white colour texture. (**A**) Tumour 11 900 from germ-line *BRCA2* mutation carrier showing AI at the *BRCA1* locus with marker D17S1322 and physical deletion of *BRCA1* by FISH. (**B**) Tumour 11 787 from germ-line *BRCA2* mutation carrier demonstrating AI at the *BRCA1* locus with marker D17S855 and no relative *BRCA1* gene copy number change by FISH. (**C**) Tumour 09 252 from germ-line *BRCA1* mutation carrier showing AI at the *BRCA2* locus with marker D13S260 and physical deletion of *BRCA2* by FISH. (**D**) Tumour 10 360 from germ-line *BRCA1* mutation carrier showing AI at the *BRCA2* locus with marker D13S260 and no *BRCA2* gene copy number change relative to 13q reference probe by FISH

© 2001 Cancer Research Campaign 

**Table 3** Summary of FISH analyses of both *BRCA1* and *BRCA2* genes in 14 sporadic breast cancers

| Sporadic cases | Interpretation of BRCA1 copy number by FISH* | Interpretation of BRCA2 copy number by FISH* |
|---|---|---|
| Case 1** | 4:2 BRCA1 deletion | 3:2 BRCA2 deletion |
| Case 2** | 4:2 BRCA1 deletion | 3:2 BRCA2 deletion |
| Case 3 | No relative BRCA1 copy number change | Monosomy of chromosome 13 |
| Case 4 | No relative BRCA1 copy number change | No relative BRCA2 copy number change |
| Case 5** | Monosomy of chromosome 17 | Monosomy of chromosome 13 |
| Case 6 | No relative BRCA1 copy number change | 3:2 BRCA2 deletion |
| Case 7** | 4:2 BRCA1 deletion | 5:3 BRCA2 deletion |
| Case 8 | No relative BRCA1 copy number change | 3:2 BRCA2 deletion |
| Case 9 | No relative BRCA1 copy number change | No relative BRCA2 copy number change |
| Case 10 | 2:1 BRCA1 deletion | No relative BRCA2 copy number change |
| Case 11 | No relative BRCA1 copy number change | No relative BRCA2 copy number change |
| Case 12 | 2:1 BRCA1 deletion | No relative BRCA2 copy number change |
| Case 13 | No relative BRCA1 copy number change | No relative BRCA2 copy number change |
| Case 14 | No relative BRCA1 copy number change | No relative BRCA2 copy number change |

*Deletion was defined if the copy number ratio (*BRCA1* gene copy number signals/chromosome 17 centromere signals or *BRCA2* gene copy number signals/*ETB* gene copy number signals) was 0.80 or less. **Concomitant loss of *BRCA1* and *BRCA2*.

(Ca 12 224). In Ca 12 224, *ETB* copy number loss was present only in approximately 50% of the tumour cells (*ETB* gene copy number average 2.50) suggesting an interstitial deletion of *BRCA2* gene in a subpopulation. Thus, loss of at least one copy of the *BRCA2* gene was present in 53% (9/17) of the *BRCA1* tumours.

All but one of the informative *BRCA1* tumours showing change in the relative *BRCA2* gene copy number showed also AI at the *BRCA2* locus (Figure 1, Table 1). 7 out of 17 (41%) of the *BRCA1* tumours did not reveal any relative *BRCA2* copy number change, although 2 of them (i.e. 2 out of 4 informative cases) showed AI of *BRCA2* (Table 1). One tumour (1/17; 6%) showed a copy number gain of the *BRCA2* gene but this tumour was not available for AI analysis (Table 1).

5 of the available 6 *BRCA2* tumours (83%) showed AI at the *BRCA1* locus (Figure 1, Table 2). All the tumours were also analysed by FISH, and 4 of them (4/8; 50%) showed a physical deletion of the *BRCA1* gene (Figure 1, Table 2). All the informative cases with deletion of *BRCA1* showed AI at the *BRCA1* locus (Figure 1, Table 2). 4 of 8 (50%) tumours revealed no relative *BRCA1* copy number change, yet 2 of these cases showed AI of *BRCA1* (Figure 1, Table 2).

### Sporadic breast tumours

14 unselected primary sporadic breast cancers were analysed for both *BRCA1* and *BRCA2* gene copy number changes by FISH. Physical deletion of *BRCA1* was detected in 6 cases (6/14, 43%). Loss of *BRCA2* was present in 7 cases (7/14, 50%). The concomitant deletion of both the *BRCA* genes was detected by FISH in only 4 tumour samples (4/14, 29%). FISH data of the sporadic tumours are summarised in Table 3.

### DISCUSSION

In the present study, we have studied *BRCA1* copy number changes and AI in *BRCA2* mutation tumours and vice versa. Only one study has been published previously on concomitant allelic loss of *BRCA1* and *BRCA2* in hereditary breast cancer. It involved 7 *BRCA1*-linked breast cancers, which showed combined LOH at *BRCA1/2* loci at high level (Kelsell et al, 1996). Unfortunately, due to low incidence of *BRCA* mutation tumours, studies of BRCA1/2 tumour features have been complicated by small sample size. Nevertheless, we were here able to study a reasonable number of *BRCA1* cases and extend the study to concern also *BRCA2* tumours. Our results showed a high prevalence (73% in *BRCA1* tumours, Table 1;67% in *BRCA2* tumours, Table 2) of combined AI of *BRCA* genes in both *BRCA1/2* tumours.

Taken together both *BRCA1* and *BRCA2* tumours available for AI analyses, concomitant allelic loss were detected in 12 (71%) out of 17 cases. In contrast, the set of sporadic breast cancer showed loss of both *BRCA* genes by FISH only in 4 (29%) out of 14 tumours (Table 3). We have shown previously that AI of both *BRCA* genes in sporadic breast cancer results mainly from physical deletion of the *BRCA* genes, which is detectable by FISH. Therefore, we think that it is possible to compare hereditary AI data with FISH data from sporadic tumours. When the frequency of concomitant loss of *BRCA1/2* genes was statistically compared between hereditary and sporadic tumours, a significant difference between these two groups was found (Pearson $\chi^2 = 5.43$; $P < 0.02$). Sporadic breast cancers reported in literature also have shown combined LOH of *BRCA1* and *BRCA2* at lower frequency (47% in Kelsell et al, 1996; 32% in Silva et al, 1999) than in the hereditary tumours analysed here. In sporadic cancers, LOH/AI has frequently been seen at either *BRCA1* or *BRCA2* locus, at 17q21 (24–38%) or 13q12–13 (18–63%), respectively (Nagai et al, 1994; Hamann et al, 1996; van den Berg et al, 1996; Niederacher et al, 1997; Phelan et al, 1998). However, controversy exists whether AI/LOH only at the single *BRCA* locus is clinically significant in sporadic tumours (Beckmann et al, 1996, Bieche et al, 1997; Silva et al, 1999).

Our results imply that combined AI at the *BRCA* loci might reflect a common pathway in tumour progression of hereditary breast cancers. In contrast, BRCA1/2 were concomitantly affected only in a minority of sporadic breast cancers, which further suggests that concomitant somatic loss of *BRCA* genes is a typical feature of hereditary and not sporadic breast tumours.

Comparison of FISH and AI data makes it possible to distinguish whether allelic imbalance is due to a physical deletion or whether it is due to other genetic mechanisms. In general, *BRCA*

 *© 2001 Cancer Research Campaign*

copy number changes and AI were in good agreement. However, in some cases AI was detected in the absence of actual gene copy number loss suggesting that deletion does not always explain AI. In theory, illegitimate homologus mitotic recombination could promote AI without any actual gene copy number losses, which are detected by FISH. However, whether these findings are truly linked to *BRCA* mutation tumours, requires further studies.

### ACKNOWLEDGEMENTS

Päivi Järvinen and Sari Toivola from the Laboratory of Cancer Biology are thanked for their technical assistance. Supported by: Finnish Academy of Sciences, Satakunta Cultural Foundation, Pirkanmaa Cancer Foundation, Finnish Cancer Society, Sigrid Juselius Foundation, Emil Aaltonen Foundation, Finnish Medical Foundation, Medical Research Fund of Tampere University Hospital.

### REFERENCES

Albertsen HM, Smith SA, Mazoyer S, Fujimoto E, Stevens J, Williams B, Rodriguez P, Cropp CS, Slijepcevic P, Carlson M, Robertson M, Bradley P, Lawrence E, Harrington T, Sheng ZM, Hoopes R, Sternberg N, Brothman A, Callahan R, Ponder BAJ and White R (1994) A physical map and candidate genes in the BRCA1 region on chromosome 17q12–21. *Nat Genet* **7**: 472–479

Beckman MW, Picard F, An HX, van Royen CRC, Dominik SI, Mosny DS, Schnürch HG, bender HG and Niederacher D (1996) Clinical impact of detection of loss of heterozygosity of BRCA1 and BRCA2 markers in sporadic breast cancer. *Br J Cancer* **73**: 1220–1226

Bieche I, Nogues C, Rivoilan S, Khodja A, Latil A and Lidereau R (1997) Prognostic value of loss of heterozygosity at BRCA2 in human breast carcinoma. *Br J Cancer* **76**: 1416–1418

Collins N, McManus R, Wooster R, Mangion J, Seal S, Lakhani SR, Ormiston W, Daly PA, Ford D, Easton DF and Stratton MR (1995) Consistent loss of the wild type allele in breast cancers from a family linked to the BRCA2 gene on chromosome 13q12–13. *Oncogene* **10**: 1673–1675

Gudmundsson J, Johannesdottir G, Bergthorsson JT, Arason A, Ingvarsson S, Egilsson V and Barkardottir RB (1995) Different tumour types from BRCA2 carriers show wild-type chromosome deletions on 13q12–q13. *Cancer Res* **55**: 4830–4832

Gyapay G, Morissette J, Vignal A, Dib C, Fizames C, Millasseau P, Marc S, Bernardi G, Lathrop M and Weissenbach J (1994) The 1993–94 Genethon human genetic linkage map. *Nat Genet* **7**: 246–339

Håkansson S, Johannsson O, Johansson U, Sellberg G, Loman N, Gerdes AM, Holmberg E, Dahl N, Pandis N, Kristoffersson U, Olsson H and Borg A (1997) Moderate frequency of BRCA1 and BRCA2 germ-line mutations in Scandinavian familial breast cancer. *Am J Hum Genet* **60**: 1068–1078

Hamann U, Herbold C, Costa S, Solomayer EF, Kaufmann M, Bastert G, Ulmer HU, Frenzel H and Komitowski D (1996) Allelic imbalance on chromosome 13q: evidence for the involvement of BRCA2 and RB1 in sporadic breast cancer. *Cancer Res* **56**: 1988–1990

Johannsson O, Ostermeyer EA, Hakansson S, Friedman LS, Johansson U, Sellberg G, Brondum-Nielsen K, Sele V, Olsson H, King MC and Borg A (1996) Founding BRCA1 mutations in hereditary breast and ovarian cancer in southern Sweden. *Am J Hum Genet* **58**: 441–450

Kelsell DP, Spurr NK, Barnes DM, Gusterson B and Bishop DT (1996) Combined loss of BRCA1/BRCA2 in grade 3 breast carcinomas. *Lancet* **347**: 1554–1555

Kerangueven F, Noguchi T, Coulier F, Allione F, Wargniez V, Simony-Lafontaine J, Longy M, Jacquemier J, Sobol H, Eisinger F and Birnbaum D (1997) Genome-wide search for loss of heterozygosity shows extensive genetic diversity of human breast carcinomas. *Cancer Res* **57**: 5469–5474

Miki Y, Swensen J, Shattuck-Eidens D, Futreal PA, Harshman K, Tavtigian S, Liu Q, Cochran C, Bennett LM, Ding W, Bell R, Rosenthal J, Hussey C, Tran T, McClure M, Frye C, Hattier T, Phelps R, Haugen-Strano A, Katcher H, Yakumo K, Gholami Z, Shaffer D, Stone S, Bayer S, Wray C, Bodgen R, Dayananth P, Ward J, Tonin P, Narod S, Bristow PK, Norris FH, Helvering L, Morrison P, Rosteck P, Lai M, Barrett JC, Lewis C, Neuhausen S, Cannon-Albright L, Goldgar D, Wiseman R, Kamb A and Skolnick MH (1994) A strong candidate for the breast and ovarian cancer susceptibility gene BRCA1. Science **266**: 66–71

Nagai MA, Yamamoto L, Salaorni S, Pacheco MM, Brentani MM, Barbosa EM, Brentani RR, Mazoyer S, Smith SA, Ponder BA and Mulligan LM (1994) Detailed deletion mapping of chromosome segment 17q12–21 in sporadic breast tumours. *Genes Chromosomes Cancer* **11**: 58–62

Neuhausen SL and Marshall CJ (1994) Loss of heterozygosity in familial tumours from three BRCA1-linked kindreds. *Cancer Res* **54**: 6069–6072

Niederacher D, Picard F, van Roeyen C, An HX, Bender HG and Beckmann MW (1997) Patterns of allelic loss on chromosome 17 in sporadic breast carcinomas detected by fluorescent-labeled microsatellite analysis. *Genes Chromosomes Cancer* **18**: 181–192

Phelan CM, Borg A, Cuny M, Crichton DN, Baldersson T, Andersen TI, Caligo MA, Lidereau R, Lindblom A, Seitz S, Kelsell D, Hamann U, Rio P, Thorlacius S, Papp J, Olah E, Ponder B, Bignon YJ, Scherneck S, Barkardottir R, Borresen-Dale AL, Eyfjord J, Theillet C, Thompson AM, Devilee P and Larsson C (1998) Consortium study on 1280 breast carcinomas: allelic loss on chromosome 17 targets subregions associated with family history and clinical parameters. *Cancer Res* **58**: 1004–1012

Silva JM, Gonzalez R, Provencio M, Dominguez G, Garcia JM, Gallego I, Palacios J, Espana P and Bonilla F (1999) Loss of heterozygosity in BRCA1 and BRCA2 markers and high-grade malignancy in breast cancer. *Breast Cancer Res Treat* **53**: 9–17

Smith AA, Easton DF, Evans DGR and Ponder BAJ (1992) Allele losses in the region 17q12–21 in familial breast and ovarian cancer involve the wild-type chromosome. *Nat Genet* **2**: 128–131

Staff S, Nupponen NN, Borg A, Isola JJ and Tanner MM (2000) Multiple copies of mutant BRCA1 and BRCA2 alleles in breast tumours from germ-line mutation carriers. *Genes Chromosomes Cancer* **28**: 432–442

Szabo CI and King M-C (1997) Population genetics of BRCA1 and BRCA2. *Am J Hum Genet* **60**: 1013–1020

Tanner MM, Karhu RA, Nupponen NN, Borg A, Baldetorp B, Pejovic T, Ferno M, Killander D and Isola JJ (1998) Genetic aberrations in hypodiploid breast cancer: frequent loss of chromosome 4 and amplification of cyclin D1 oncogene. *Am J Pathol* **153**: 191–199

Tavtigian SV, Simard J, Rommens J, Couch F, Shattuck-Eidens D, Neuhausen S, Merajver S, Thorlacius S, Offit K, Stoppa-Lyonnet D, Belanger C, Bell R, Berry S, Bogden R, Chen Q, Davis T, Dumont M, Frye C, Hattier T, Jammulapati S, Janecki T, Jiang P, Kehrer R, Leblanc JF, Mitchell JT, McArthur-Morrison J, Nguyen K, Peng Y, Samson C, Schroeder M, Snyder SC, Steele L, Stringfellow M, Stroup C, Swedlund B, Swensen J, Teng D, Thomas A, Tran T, Tran T, Tranchant M, Weaver-Feldhaus J, Wong AKC, Shizuya H, Eyfjord JE, Cannon-Albright L, Labrie F, Skolnick MH, Weber B, Kamb A and Goldgar DE (1996) The complete BRCA2 gene and mutations in chromosome 13q-linked kindreds. *Nat Genet* **12**: 333–337

van den Berg J, Johansson O, Håkansson S, Olsson H and Borg Å (1996) Allelic loss at chromosome 13q12–q13 is associated with poor prognosis in familial and sporadic breast cancer. *Br J Cancer* **74**: 1615–1619

Welcsh PL, Owens KN and King MC (2000) Insights into the functions of BRCA1 and BRCA2. *Trends Genet* **16**: 69–74

Wooster R, Neuhausen SL, Mangion J, Quirk Y, Ford D, Collins N, Nguyen K, Seal S, Tran T, Averill D, Fields P, Marshall G, Narod S, Lenoir GM, Lynch H, Feunteun J, Devilee P, Cornelisse CJ, Menko FH, Daly PA, Ormiston W, McManus R, Pye C, Lewis CM, Cannon-Albright LA, Peto J, Ponder BAJ, Skolnick MH, Easton DF, Goldgar DE and Stratton MR (1994) Localization of a breast cancer susceptibility gene, BRCA2, to chromosome 13q12–13. *Science* **265**: 2088–2090

*© 2001 Cancer Research Campaign*
